# Cardiovascular effects of hypertonic lactate solutions: acid–base or metabolic cause, or both?

**DOI:** 10.1186/s13054-025-05463-y

**Published:** 2025-05-30

**Authors:** Pedro J. Freire Jorge, Rohan Boer, František Duška, Maarten M. W. Nijsten, Micah L. A. Heldeweg

**Affiliations:** 1 Department of Critical Care, University Medical Center Groningen, Groningen, The Netherlands; 2 Department of Anesthesiology, Amsterdam University Medical Centers, Amsterdam, The Netherlands; 3 Department of Anesthesia and Intensive Care Medicine, The Third Faculty of Medicine, Charles University and FNKV University Hospital, Šrobárova 1150/50, 100 34 Prague, Czech Republic

**Keywords:** Acid–base, Buffer, Carbon dioxide, Respiratory, Metabolic, Lactate, Saline

It was a pleasure to read the recent publication in *Critical Care* on the cardiovascular effects of lactate [1]. The authors conducted a randomized comparison between the infusion of hypertonic lactated solution and hypertonic saline solution and their effects on echocardiography measurements. The authors equalized the osmolarity of the solutions to ensure that there were no differences in volume expansion between groups—as evidenced by the preload metrics. The authors assert that the observed hemodynamic differences were primarily attributable to the infusion of hypertonic lactate i.e., lactate anion.

Increasing evidence supports lactate as a valuable metabolic intermediate and signaling molecule, highlighting its potential future therapeutic role. However, before concluding on the positive effects of exogenous hypertonic lactate solutions, we would like to discuss additional acid–base and metabolic considerations that will contribute in moving the discourse and future investigations forward.

The first point concerns the confounding acid–base effects secondary to the administration of hypertonic sodium-lactate and sodium-chloride. In the current study, the sodium-lactate and sodium-chloride groups demonstrated significant and substantial differences in pH, chloride, bicarbonate, lactate, and potassium levels. Although the authors stated that there is no hyperchloremic acidosis, we calculated an average difference in the standard base excess of − 7.4 mmol/L (−2.1 versus + 5.3) between the groups. The acid–base difference between groups is virtually entirely—barring a minimal amount of unmeasured anion in the sodium-lactate group—explained by their relative changes in chloride (see supplemental material for full calculation).

As lactate is metabolized, the strong ion difference in the sodium-lactate group rises, whereas the strong ion difference in the sodium-chloride group decreases due to hyperchloremia. This is a problematic confounder because a hypochloremia with metabolic alkalosis has a positive inotropic effect, whereas a hyperchloraemia with metabolic acidosis causes myocardial depression [2]. Thus, the different cardiovascular effects (reflected in a different stroke volume) between these groups may well be attributed to the electrolyte or acid-base properties of the respective solutions [2]. In fact, previous experiments in hemorrhaging canines also demonstrated the superior cardiovascular effects of hypertonic fluids with either acetate or lactate, versus unbalanced hypertonic saline. The metabolization of acetate, another organic anion, also induces alkalosis in a manner similar to that of lactate. The authors found that acetate may have a superior resuscitative profile due to its strong intrinsic vasodilatory properties and capacity to persistently restore oxygen consumption [3]. Moreover, another study in hemorrhaging dogs showed that infused acetate was rapidly metabolized, whereas equivalent lactate loads exceeded the capacity for metabolism and caused a progressive accumulation, worsening the acid–base status and potentially the hemodynamics [4]. These effects are also replicated in clinical studies that compared infusion of saline versus non-lactate balanced crystalloids during surgery and reported similar adverse hemodynamic effects of saline [5]. This suggests that expanding volume with fluids with a higher strong ion difference, rather than a specific energetic or signaling profile of lactate, may be the factor driving differences in cardiovascular effects.

The second point concerns the administration of 50% sodium L-lactate and 50% sodium D-lactate hypertonic sodium lactate. We agree with the recent comment by Stevic and colleagues that infusion of racemic lactate appears suboptimal in this research design [6]. First, L-lactate is the primary end-product of glycolysis and a key energy substrate, whereas D-lactate is a minor byproduct of methylglyoxal metabolism, present at much lower physiological concentrations [7]. High levels of D-lactate may impair L-lactate uptake and metabolism (in myocardial cells) by competing for monocarboxylate transporters and accumulating intracellularly, potentially inhibiting full mitochondrial oxidation of L-lactate [8] Therefore, administering a pure L-lactate solution may enhance myocardial energy delivery more effectively than a racemic mixture. Second, the use of pure L-lactate also facilitates a more comprehensive acid–base understanding. Since D-lactate is not detected by conventional blood gas analyzers, the unmeasured D-lactate level in the current study could fully explain the remaining minimal difference in strong ions and anion gap missing from the acid–base calculations (see supplemental materials for full calculation) [9]. Furthermore, the original authors propose a direct alkalinizing effect of lactate independent of metabolism in their reply in *Critical Care* [10], but this interpretation does not align with the Stewart’s physicochemical model. Both sodium and lactate (whether D- or L-form) are fully dissociated ions in solution and thus contribute to the strong ion difference. Because they are present in equimolar amounts, the infused solutions have a net strong ion difference of zero. Therefore, the *direct* effect of the infused solutions, according to the Stewart model of acid–base, is acidification [10, 11]. The *indirect* alkalinizing effect of the infused solution is dependent on the subsequent removal of (D- or L-)lactate from extracellular fluid (i.e. through metabolization), which increases the strong ion difference. Thus, altered or uncertain metabolism of D-lactate may lead to its accumulation, potentially disrupting metabolism and acid–base balance, as well as complicating its accurate interpretation.

Many experimental and clinical investigations point to the beneficial effects of exogenous L-lactate administration, even in hypertonic concentrations. L-Lactate functions as a highly relevant metabolic intermediate in glycometabolism and an important signaling molecule in various tissues, not solely the liver and kidneys [12]. While acetate-balanced solutions promote alkalosis, enhance microcirculation and cardiac function [13], L-lactate surpasses other organic anions in physiological relevance and is the dominant circulating energetic metabolite [12]. A landmark study demonstrated that L-lactate has a systemic turnover flux five times higher than that of acetate and 23 times higher than citrate, underscoring its central role in shuttling energy between cells [14]. Superimposed on its properties as a metabolic fuel, lactate functions as a signaling molecule capable of modulating many biological (including cardiovascular) processes. In healthy volunteers with substantial cardiac reserves, such as in the current study, an increased circulating lactate load could also serve as a signal that an increased cardiac output is required. However, to truly investigate lactate’s unique benefits, future studies must compare solutions of pure L-lactate with solutions matched for both osmolarity and strong ion difference, for example a matched sodium-bicarbonate or sodium-acetate solution.

The evidence for hypertonic lactate solutions in different clinical settings is compelling. Whether an acid–base or a metabolic effect, or both, is the cause of improved cardiovascular function remains to be discovered.

## Supplementary Information

Additional file1

## Data Availability

Not applicable.
