# Supplementary material for: Cardiovascular effects of hypertonic lactate solutions: acid–base or metabolic cause, or both?
Source: Crit Care. 2025 May 30;29:217. doi: 10.1186/s13054-025-05463-y (PMC12123888; doi:10.1186/s13054-025-05463-y)
Supplement: Supplementary file 1 — Additional file1 [file 13054_2025_5463_MOESM1_ESM.docx]

**Supplemental materials**

The calculation of standard base excess was performed using the widely established standard base excess equation [1]:

$$\boldsymbol{Standard base excess=}\left( \boldsymbol{HC}\boldsymbol{O}_{\boldsymbol{3}}^{\boldsymbol{-}}\boldsymbol{-24.8} \right)\boldsymbol{+}\left( \boldsymbol{pH-7.40} \right)\boldsymbol{\cdot16.2}$$

The laboratory data by Berg-Hansen is reported as mean and standard deviation, which allows us to assume a normal distribution and conduct calculations with the means.

For the LAC group after treatment standard base excess can be calculated as follows:

$$\left( \boldsymbol{29.6-24.8} \right)\boldsymbol{+}\left( \boldsymbol{7.43-7.40} \right)\boldsymbol{\cdot16.2=5.3}$$

For the SAL group after treatment standard base excess can be calculated as follows:

$$\left( \boldsymbol{23.5-24.8} \right)\boldsymbol{+}\left( \boldsymbol{7.35-7.40} \right)\boldsymbol{\cdot16.2=-2.1}$$

The difference in base excess after treatment is therefore -7.4 mmol/L for the SAL group. This signifies an acid (or anion) load of 7.4 mmol/L in the SAL group. The cause may be related to differences in strong ions between the groups after treatment and can be assessed by partitioning the strong ion differences into the individual electrolyte contributions.

*Supplemental table 1. Differences in average SAL and LAC electrolyte values after treatment and their relative contribution to strong ion difference*

|  | LAC | SAL | Relative SID contribution for SAL |
| --- | --- | --- | --- |
|  |  |  |  |
| Cations (positive contributions to SID) |  |  |  |
| Sodium | 146 | 146 | 0 |
| Potassium | 3.6 | 4.1 | +0.5 |
| Calcium | 1.15 | 1.18 | +0.03 |
|  |  |  |  |
| Anions (negative contributions to SID) |  |  |  |
| Chloride | 107 | 117 | -10 |
| L-Lactate | 2.5 | 0.9 | +1.6 |
|  |  |  |  |
| **Final** |  |  | **-7.9** |
| **Base Excess** | **5.3** | **-2.1** | **-7.4** |
| *Unexplained difference* |  |  | *-0.5* |

This shows that the base excess is almost fully explained by the increase in chloride in the SAL group. The remaining unexplained charges may be caused by an unmeasured anion in the LAC group or an unmeasured cation in the SAL group. Considering the nature of the experiment it is unlikely that these charges may be explained by unreported minor strong or weak ions such as albumin, phosphate, or magnesium. However, since the experiment was performed with 50% racemic lactate, a difference in unmeasured D-lactate, is entirely plausible. Last, considering both the lower *in vivo* non-carbonic buffer power and its interindividual variance, it is likely that D-lactate plays a larger role than only 0.5 mmol/L[1,2]. Performing the calculations with the *in vivo* buffer coefficient leads to a difference of 1-2 mmol/L.

This is presence of unmeasured D-lactate is further supported by calculating the estimated differences in the unmeasured anion gap using the available cations and anions:

$$\left( \boldsymbol{Sodium+Potassium+Calcium} \right)\boldsymbol{-}\left( \boldsymbol{Chloride+Bicarbonate+ Lactate} \right)$$

For the LAC group after treatment the calculation is as follows:

$$\left( \boldsymbol{146+3.6+1.15} \right)\boldsymbol{-}\left( \boldsymbol{107+29.6+ 2.5} \right)\boldsymbol{=}\boldsymbol{11.65}$$

For the SAL group after treatment the calculation is as follows:

$$\left( \boldsymbol{146+4.1+1.18} \right)\boldsymbol{-}\left( \boldsymbol{117+23.5+ 0.9} \right)\boldsymbol{=}\boldsymbol{9.88}$$

The final estimation of unmeasured anion difference is 1.72 mmol/L, potentially attributable to D-lactate. This is in accordance with the 1.7 mmol/L rise in L-lactate seen in the LAC group from racemic sodium lactate with 50% L-lactate and 50% D-lactate.

**References**

1. Heldeweg MLA, Berend K, Schober P, Duška F. Understanding the Acid-Base Response to Respiratory Derangements: Finding, and Clinically Applying, the In Vivo Base Excess. Crit Care Explor. 2024 Dec 16;6(12):e1191. doi: 10.1097/CCE.0000000000001191. PMID: 39691548; PMCID: PMC11651497.
2. Lu J, Zello GA, Randell E, Adeli K, Krahn J, Meng QH. Closing the anion gap: contribution of D-lactate to diabetic ketoacidosis. Clin Chim Acta. 2011 Jan 30;412(3-4):286-91. doi: 10.1016/j.cca.2010.10.020. Epub 2010 Oct 29. PMID: 21036159.
